# Experimental evidence of new tetragonal polymorphs of silicon formed through ultrafast laser-induced confined microexplosion

**DOI:** 10.1038/ncomms8555

**Published:** 2015-06-29

**Authors:** L. Rapp, B. Haberl, C.J. Pickard, J.E. Bradby, E.G. Gamaly, J.S. Williams, A.V. Rode

**Affiliations:** 1 Laser Physics Centre, Research School of Physics and Engineering, The Australian National University, Canberra, Australian Capital Territory 0200, Australia; 2 Electronic Materials Engineering, Research School of Physics and Engineering, The Australian National University, Canberra, Australian Capital Territory 0200, Australia; 3 Department of Physics and Astronomy, University College London, London WC1E 6BT, UK

## Abstract

Ordinary materials can transform into novel phases at extraordinary high pressure and temperature. The recently developed method of ultrashort laser-induced confined microexplosions initiates a non-equilibrium disordered plasma state. Ultra-high quenching rates overcome kinetic barriers to the formation of new metastable phases, which are preserved in the surrounding pristine crystal for subsequent exploitation. Here we demonstrate that confined microexplosions in silicon produce several metastable end phases. Comparison with an *ab initio* random structure search reveals six energetically competitive potential phases, four tetragonal and two monoclinic structures. We show the presence of bt8 and st12, which have been predicted theoretically previously, but have not been observed in nature or in laboratory experiments. In addition, the presence of the as yet unidentified silicon phase, Si-VIII and two of our other predicted tetragonal phases are highly likely within laser-affected zones. These findings may pave the way for new materials with novel and exotic properties.

## Abstract (web summary)

Ordinary materials can transform into exotic phases with new crystal structures at high pressure and temperature. Here, the authors demonstrate metastable phases of silicon, created by confined microexplosions initiated by ultrafast laser pulses, that are preserved for further utilisation.

## Graphical abstract

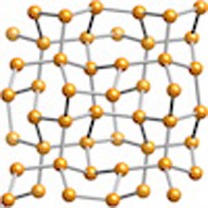

The traditional tool for high-pressure laboratory synthesis of new materials under static (near-equilibrium) conditions is the diamond-anvil cell (DAC)^1^,^2^. For example, in silicon 13 different pressure-induced crystalline phases have been reported to date at pressures up to 250 GPa (ref. 3). Such transitions are not fully reversible during pressure release at ambient temperature as a result of strong kinetic effects in the Si system^3^. This leads to several metastable structures on full pressure release such as the r8 and bc8 phases^3^,^4^,^5^. These metastable Si polymorphs are predicted to exhibit a wide range of characteristics, from semiconducting to semimetallic, while other predicted metastable phases such as the st12 phase, which is yet to be observed at ambient pressure, is likely to be superconducting^4^,^6^. This signifies not only their scientific interest but also their likely technological importance. This success of static, near-equilibrium methods in producing interesting metastable silicon phases begs the question of whether a wider range of novel materials is possible by accessing ultra-rapid non-equilibrium conditions of high pressure and temperature. In fact, such short timescale, non-equilibrium conditions can be achieved in shock wave (SW) studies that yield dynamic compression at the front of an intense SW driven by super-intense lasers^7^,^8^,^9^,^10^, high-energy explosives^11^ or even in a combination with static methods^12^,^13^. The predominant feature of both these static and dynamic methods is that an initially crystalline arrangement of atoms is subjected to either slowly increased pressure in the DAC or rapidly applied SW pressure. Under such conditions, the atoms are forced into closer interatomic positions and induce transformation from one crystalline state into another more dense state. However, the natural pressure limit of the Young's modulus of diamond in the static DAC experiments limits the maximum pressure achievable. Equally, the very short timescale of tens of nanoseconds before expansion and ablation limits SW studies severely, particularly the observation of new phases. To a large extent we have overcome these difficulties by applying an ultrafast laser-induced confined microexplosion within the bulk as a new, alternative way to form novel materials using Si as a technologically interesting example.

Ultrashort laser-induced confined microexplosions generate extremely high pressure and high temperature conditions^14^,^15^,^16^,^17^,^18^,^19^, potentially offering a more favourable route to new metastable end phases. A confined microexplosion induced by femtosecond-range (∼100 fs) laser pulses tightly focused inside the bulk of transparent matter to intensities of ∼10^14^ W cm^−2^ offers the prospect of creating simultaneously TPa pressures and temperatures above 10^5^ K in table-top experiments. Such conditions result in ∼100 nJ of absorbed energy and at an energy density of several MJ cm^−3^, higher than the strength of any material. At these energy densities, condensed matter is transformed into a high-entropy state of dense plasma with temperatures in the order of tens of eV and a density well above the solid state. Thus, all memory of the initial crystalline state is completely lost in this state, termed Warm Dense Matter (WDM). WDM is a non-equilibrium state of matter between solid and plasma. It can be defined as the state that is too dense to be described by weakly coupled plasma physics, yet that is too energetic to be described by condensed matter physics. In this state, the potential energy of the interaction between electrons and nuclei and the kinetic energy of electrons are of roughly the same magnitude. Diverse material structures can spontaneously organize themselves during cooling from such a hot, extremely dense plasma composed of a completely disordered mixture of elements far away from thermodynamic equilibrium^20^. Such new structures occur, as the randomized atomic configuration within the laser-produced plasma cools down isochorically to ambient temperature with an unprecedented quenching rate of up to 10^14^ K s^−1^ (refs 13, 14). Clearly, these conditions are favourable for creation of novel metastable end phases that may not be energetically accessible by any other experimental means. Such an experimental path to new structures from a completely disordered state is in close analogy to the computational Ab Initio Random Structure Searching (AIRSS) approach^21^,^22^,^23^ where the prediction of novel phases created under high pressure starts with the material in a disordered state. AIRSS calculations, for example, have led to the prediction of incommensurate aluminium with an unconventional spatial distribution of atoms and valence electrons^24^. The crucial feature of a confined microexplosion is the ability to facilitate this novel path to formation of unusual phases, which then are preserved within the bulk of a pristine crystal. This enables measurements of the properties of such new materials and, ideally, their preparation in sufficient volume for subsequent exploitation. To date, this method has been predominantly applied to transparent materials. Through a capping technique, we extend the powerful microexplosion method to non-transparent materials^25^, making it applicable for a wide range of materials such as semiconductors, metals, non-transparent oxides and many other solids.

In this study we achieve formation of new phases in Si by confined microexplosions initiated by ultrafast laser pulses. We note here that femtosecond laser pulses focused at the freestanding silicon surface without the condition of confinement have also resulted in significant material modifications, including substantial ablation, defects and stacking faults. Evidence for the metastable r8 and bc8 Si phases as well as the tetragonal Si-VIII phase, known from DAC experiments, has been reported within such laser-affected regions—see refs 26, 27, 28. Specifically, we observe, in the regions affected by the temperature and SW, at least three metastable tetragonal polymorphs of Si, two of which are synthesized for the first time. The structure of these new polymorphs is determined from comparison with energetically highly competitive structures predicted by AIRSS. In addition, our data also reveal the presence of other as yet unidentified Si phases, and we suggest possible candidates also from AIRSS.

## Results

### Confined microexplosion

In our study, a powerful ultrashort and tightly focused laser pulse is used to generate a microexplosion at the interface between a transparent amorphous silicon dioxide layer (SiO_2_) and an opaque crystalline Si substrate. The microexplosion is a complex phenomenon, which is described by a sequence of processes leading to the formation of void surrounded by a shell of compressed material where the permanent transformation of the affected material into new phases takes place^14^,^15^,^16^,^17^,^18^,^19^.

There are several stages in the process of the formation of voids and of compressed material surrounding these voids. The ultrashort laser pulse at an absorbed energy above the optical breakdown threshold generates plasma, where the energy density is up to several MJ cm^−3^, higher than the internal strength (Young's modulus) of Si and SiO_2_ (ref. 29). At the end of the laser pulse, the electron temperature reaches several tens of eV and the pressure several TPa^14^,^15^,^16^,^17^,^18^,^19^. Such hot electrons generate an electronic heat wave that propagates into the surrounding cold solid. They transfer their energy to ions by Coulomb collisions, thus transforming the heat wave into a SW. The SW propagates into the bulk and creates a rarefaction wave that, due to conservation of mass, produces a sharp density decrease in the central zone leading to the formation of a void in the laser-irradiated material. Thereafter, the heated and SW-affected material quenches quickly to ambient conditions via electronic heat conduction^10^. The presence of voids in the bulk of a laser-irradiated material is unequivocal evidence for the creation of a pressure well in excess of the Young's modulus of the material during the laser-induced microexplosion^15^,^16^. The SW-compressed and -quenched shell around the void is the area where new phases are found.

**Figure 1:**
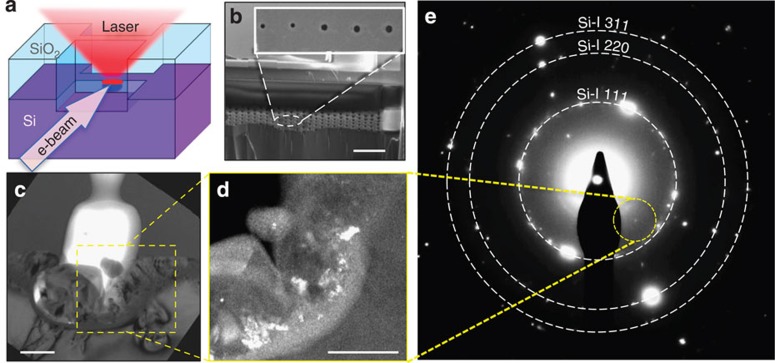
Ultrashort laser-induced confined microexplosion experiments in silicon. (**a**) Schematic depiction of a microexplosion at the Si surface under a layer of SiO_2_ in a sample cut by a focused ion beam (FIB) for electron diffraction analysis. (**b**) Scanning electron microscope (SEM) image of FIB-opened voids produced by 440-nJ fs-laser pulses focused to 95 J cm^−2^; scale bar, 10 μm (the inset shows a magnified top view of the voids located 2 μm apart). (**c**) TEM bright-field image of a void at the Si/SiO_2_ interface (the laser pulse was coming from the top); the shock-wave-modified area in Si is clearly observed under the void in SiO_2_. (**d**) A magnified dark-field TEM image of the shock-wave-affected area beneath the Si surface. Scale bars, 200 nm (**c**,**d**). (**e**) Electron diffraction pattern from the area marked in **c**. The yellow dashed circle in **e** indicates the reflections used to generate the dark-field image shown in **d**.

The permanent transformation induced by the laser can be studied using transmission electron microscopy (TEM). Therefore, cross-sections are thinned down to ∼80 nm for electron transparency using focused ion beam (FIB) milling. The geometry of such a thinned section is shown schematically in Fig. 1a. Once the Si/SiO_2_ becomes sufficiently thinned (but prior to electron transparency), the voids induced by the laser become visible (Fig. 1b). This allows for exact locating of the phase-transformed zone for further thinning followed be examination in a TEM.

### Electron diffraction patterns

TEM images clearly show the presence of a drastically laser-modified volume in Si (Fig. 1c,d). Selected area electron diffraction patterns (SADPs) taken from the modified Si area show, in addition to conventional diamond cubic Si (dc-Si), a large number of not previously observed d-spacings (Fig. 1e). These indicate the presence of several polymorphs of Si. The dark-field image taken by using three bright reflections of such newly identified tetragonal polymorphs (indicated in the diffraction pattern) confirms that these unidentified d-spacings originate indeed from crystals within the Si substrate.

The analysis of these electron diffraction patterns for phase identification is relatively complex due to the nanocrystallinity of the laser-modified area. Such nanocrystallinity is typical for pressure-induced metastable phases of Si with, for example, crystal sizes of 3 nm to a few 10 s of nm reported for bc8 formed in a DAC from single-crystal dc-Si^30^. This appears as a direct result of the large density difference (>20%) between dc-Si and any pressure-induced metallic phase of Si^3^, which results in the formation of polycrystalline material upon compression (a few 10 s of nm for metallic Si formed from dc-Si^31^). From dark-field imaging within the TEM (Fig. 1d for an example) the crystal size in our case can be estimated as ∼10–30 nm. Due to the relatively small number of crystals sampled with a selected area aperture in such thinned FIB sections, this results in a very small number of crystals that are in a positive diffraction condition (in the order of 1–3 crystals only) and thus recorded on the SADP. Consequently, our electron diffraction patterns consist neither of powder diffraction nor of single-crystal diffraction, but can be thought of as diffraction from a small number of single crystals. Hence, it is clear that no standard powder diffraction analysis or analysis for simple single-crystal diffraction can be employed in such a case. Instead, a more complex analysis was required to properly index the diffraction patterns.

In short, first, we used the calibration parameters of the TEM, consequently refined by the dc-Si reflections, to determine the d-spacings of all non-dc-observed reflections in the diffraction patterns; second, we inspected the diffraction patterns for matches with the known pressure-induced metastable phases of Si; thereafter, we matched reflections not assigned to any known Si phases to d-spacings calculated from the phases predicted by the computational search; finally, for further confirmation of the presence of a given phase, we laid a simulation of a single-crystal diffraction pattern of this phase over the SADP. This confirmed that several reflections matched the predicted phase not just in terms of d-spacings but also for angles—see Methods for details.

### Computational search

**Figure 2:**
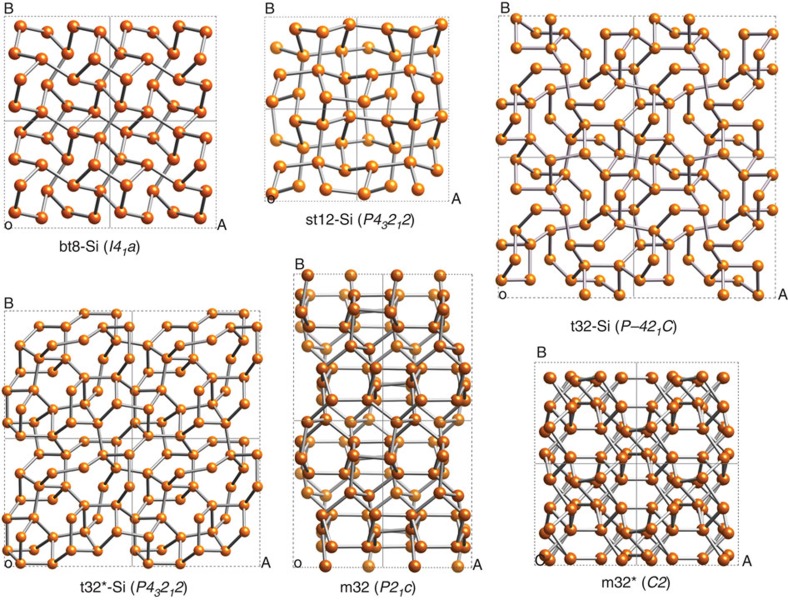
Crystal structure of new polymorphs of Si uncovered in the AIRSS searches. The lattice parameters, volumes of unit cell per atom and enthalpy differences are taken from the 0-GPa computed structures (see Table 1 and Supplementary Data 1–6). Each image is comprised of four primitive cells. The three-dimensional animations showing different viewing angles prepared with CrystalMaker are presented in the Supplementary Fig. 1.

To identify the crystal structures visible in the SADPs, we performed a thorough computational search for dense metastable Si phases using AIRSS^21^,^22^,^23^,^24^. A useful feature of AIRSS, because it is not exclusively biased towards low enthalpy configurations, is that it identifies many structures that are metastable at a given pressure, but may become thermodynamically more stable at other pressures. This makes it more effective for large pressure ranges than searching independently for optimal (stable) structures at each pressure of interest. The calculations were performed using the CASTEP code, a robust first-principles code for the prediction of material properties, based on plane waves and pseudopotentials^32^. The AIRSS uncovered the experimentally well-known bc8 (space group *Ia-3*), r8 (space group *R-3*) and the earlier predicted st12 (space group *P4*_*3*_*2*_*1*_*2*) metastable tetrahedrally bonded polymorphs^4^. It also revealed several new and very energetically competitive phases of Si, three further tetragonal phases and two monoclinic structures—see Supplementary Movies 1–6. The first tetragonal phase with space group *I4*_*1*_*/a* and 16 atoms in the conventional cell is in fact the very same structure recently predicted by Wang *et al.*^33^ who used first-principles calculations to investigate transition pathways in Si. They suggested this metastable phase as an intermediate between the metallic β-Sn structure and the well-known bc8 phase formed on decompression via a double-cell bond-rotation mechanism with a low kinetic barrier. This structure was designated as tetragonal bt8 silicon^33^. In addition, they investigated the possibility of a transition to the 12-atom tetragonal structure st12, which has previously been predicted for Si^4^,^34^, but not experimentally observed, based on the fact that it is observed in germanium^3^,^35^. However, the pathway towards st12 was considered unfavourable in Si due to a large conversion barrier of 0.21 eV (ref. 33). Finally, two further tetragonal phases (with space groups *P-42*_*1*_*c* and *P4*_*3*_*2*_*1*_*2*) and two monoclinic phases (with space groups *C2* and *P2*_*1*_*c*) with large unit cells of 32 atoms were uncovered by our search—see Supplementary Movies 1–6. Such structures are of significant interest since two experimentally known tetragonal polymorphs of Si (Si-VIII and Si-IX) possess equally large unit cells^36^, but their full structure remains unidentified. All these structures are summarized in Table 1, whereby lattice parameter, volume per atom and enthalpy (the latter relative to bc8-Si) are the values calculated at 0 GPa. For ease of further reading, the newly uncovered structures will be denominated t32, t32*, m32 and m32*. The crystal structures of these new phases uncovered using the CASTEP code are shown in Fig. 2, where four primitive cells for each structure are illustrated. Finally, the lattice parameters of the newly predicted phases bt8, st12, t32 and t32* at different pressures are additionally summarized in Table 2. Note that no strong experimental evidence for the m32 and m32* phases was obtained and they are hence omitted in Table 2.

### Experimental metastable phases

**Figure 3:**
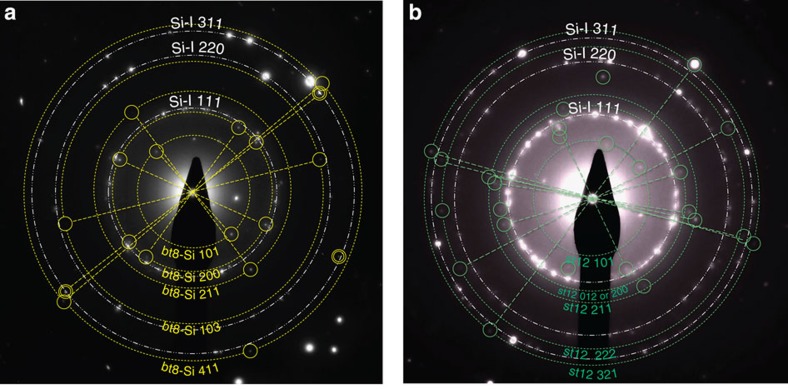
Electron diffraction patterns from tetragonal Si phases. Predominantly (**a**) bt8-Si signatures are produced by a laser fluence of 95 J cm^−2^ and (**b**) st12-Si signatures by a laser fluence of 48 J cm^−2^. In addition, in both cases evidence of further tetragonal phases such as Si-VIII and t32-Si is observed. In both SADPs the dc-Si diffraction rings used for calibration are indicated by white dash-dotted circles. The bt8-Si and st12-Si diffraction rings are depicted by yellow and light-green dotted circles, respectively. The d-spacings used for these rings are calculated from the experimental lattice parameters of bt8-Si and st12-Si. Corresponding pairs of reflections are connected by radial dashed lines to guide the eye.

Many electron diffraction patterns were investigated and representative examples from two different laser-affected sites are presented in Fig. 3. The interatomic distances measured from these SADPs are summarized in Table 3. They were reproduced in several diffraction patterns taken from different laser-modified zones.

Clearly, the majority of the reflections can be matched to dc-Si, as well as the predicted bt8-Si and st12-Si. Consequently, diffraction circles corresponding to d-spacings indicative of the bt8 phase (yellow) are indicated in Fig. 3a and of the st12 phase (green) in the Fig. 3b. These are the predominant phases in the respective SADP, but Si-VIII and potentially the tetragonal structures with 32 atoms (t32 and t32*) are also present as shown later. No evidence for the monoclinic phases was detected with only a minority (∼20%) of their d-spacings loosely attributable to observed values. Their presence can, however, not be fully excluded from further SADPs not shown here and further investigation is warranted.

From these diffraction rings indexed to bt8 and st12, respectively, the unit cell of the experimental phases was obtained through a refinement that minimized a *χ*^2^-fit of calculated d-spacings to the measured d-spacings summarized in Table 4. A good quality refinement was achieved with a total *χ*^2^ of ∼0.001 in all cases between measured d-spacings and those calculated from the refined unit cell. Note that although this *χ*^2^ is smaller than the error associated with the measurement of the respective d-spacings, it is still quoted to allow for comparison of the quality of the fit (see Table 4). For bt8-Si, unit cells of *a=*6.64* *Å, *c=*6.43 Å and *a=*6.68 Å, *c=*6.32 Å were obtained for SADP(a) and SAPD(b), respectively. For st12-Si, *a=*5.69 Å, *c=*6.77 Å and *a=*5.67 Å, *c=*6.79 Å were obtained for SADP(a) and SAPD(b), respectively. In the st12 case, these lattice parameters are consistently (∼0.01–0.04 Å) larger than the calculated unit cell at 1 GPa. This indicates that this phase is at ambient pressure and little residual stress remains. In contrast, in the case of bt8 the experimental *c*-value is consistently smaller than the calculated unit cell at 1 GPa (0.03 Å) and more comparable to the calculated unit cell at 2 GPa. This would appear to be indicative of high residual stresses. However, the *a*-value is larger or close to the unit cell calculated at 1 GPa. Therefore, we suggest that bt8 may be, equally to st12, at ambient pressure, but that its unit cell is somewhat strained. These findings are consistent with the fact that investigation of the laser-modified zones by TEM required thinning down to 100 nm of thickness. Such a thinned section will not maintain (much) residual stress and hence lattice parameters indicative of ambient pressure were to be expected.

**Figure 4:**
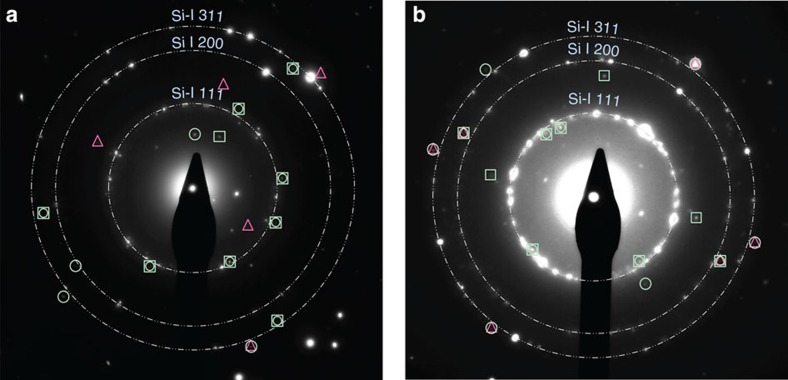
(**a**,**b**) Additional reflections in the electron diffraction pattern. The evidence of further tetragonal phases such as Si-VIII marked with triangles, t32-Si marked with squares and t32* marked with circles, all present in the same electron diffraction patterns as in (**a**) and in (**b**).

Figure 4 presents the same diffraction patterns as in Fig. 3 above, but indicating diffraction spots of three further tetragonal phases, namely Si-VIII (with its experimental d-spacings taken from ref. 36) and the two new t32 phases (t32-Si and t32*-Si) calculated with density functional theory (DFT)—see Table 2. No diffraction pairs were indicated for Si-VIII and t32/t32*-Si in the image due to the small number of reflection spots assigned to these phases. Consequently, no matching of diffraction from these phases with corresponding single-crystal diffraction was possible, and phase assignment was solely conducted via comparison of d-spacings. No experimental lattice parameters could be calculated for Si-VIII due to open questions regarding its structure (it cannot be predicted by structure search, for example). Nonetheless, the average deviation of our observed reflection spots assigned to Si-VIII compared with previously determined X-ray diffraction data is 0.002 Å for SADP(a) and 0.003 Å for SADP(b). For t32-Si, the refinement yielded *a*=9.39 Å for both SADPs with *χ*^2^=0.002 and 0.0004 for (a) and (b), respectively. The refinement was slightly worse for t32*-Si and resulted in *a*=9.45 Å in both SADPs with *χ*^2^=0.003 and 0.002 for (a) and (b), respectively. In both cases, the experimental *c* could not be calculated since only *hk0* reflections were assigned to these phases. This is also summarized in Table 4, where we state the value for *c* from the calculations in the absence of refinement from our experimental data. These observations for Si-VIII and t32-Si suggest that both phases also exist at 0 GPa pressure, consistent with findings for bt8-Si and st12-Si. In contrast, lattice parameters determined for t32*-Si appear to be slightly larger than those computed at 0 GPa. This could be a result of a strained unit cell or could alternatively suggest that t32*-Si is a less likely candidate than t32-Si.

## Discussion

Our combined experimental results and theoretical analysis show that two of the tetragonal Si phases created are indeed bt8 and st12. Both phases are thermodynamically stable at ambient temperature, as confirmed by the observation of similar reflections in SADPs taken more than a year after the initial observation. Many additional diffraction spots can be assigned to the tetragonal Si-VIII and the potential candidate structures t32 and t32*. Si-VIII has been previously observed in a single experimental DAC study following very rapid unloading^36^ and also after irradiation of a Si surface with an ultrashort pulsed laser in the absence of confinement^28^. The formation of these tetragonal polymorphs, reliably repeatable in a large number of microexplosion experiments, is evidence that Si has undergone pressure-induced transitions into the realm of the metallic high-pressure phases that are conventionally formed above 11 GPa (ref. 3). A number of reflections in our diffraction patterns (Figs 3 and 4) remain unexplained and suggest the presence of at least one additional entirely new and as yet unpredicted phase. We further note that we compared our data also against the predicted t12 structure^37^, but no good match was obtained.

The estimates for the maximum temperature and pressure during the fs-pulse-driven microexplosion are given in the Supplementary Note 1. A maximum electron temperature of around 10^5^ K and pressure of 10 TPa in the dense plasma are reached at the end of the laser pulse. The subsequent thermal transfer to the surrounding lattice and fast quenching occurs at the rate of 10^14^ K s^−1^, while the depressurization occurs at an average rate of ∼10^10^ GPa s^−1^. Such conditions are far from thermodynamic equilibrium compared with the typical, near-equilibrium (de)pressurization rates of previous DAC experiments. We might therefore expect that the phase transformation pathways on depressurization in our microexplosion case are also different. The near-equilibrium DAC studies have revealed the metastable crystalline r8 and bc8 phases (space groups *R-3* and *Ia-3*, respectively) on pressure release from the metallic β-Sn phase and an additional (lonsdaleite) hexagonal diamond (hd) structure on annealing^5^. Intriguingly, we do not observe any of these phases under our microexplosion case.

**Figure 5:**
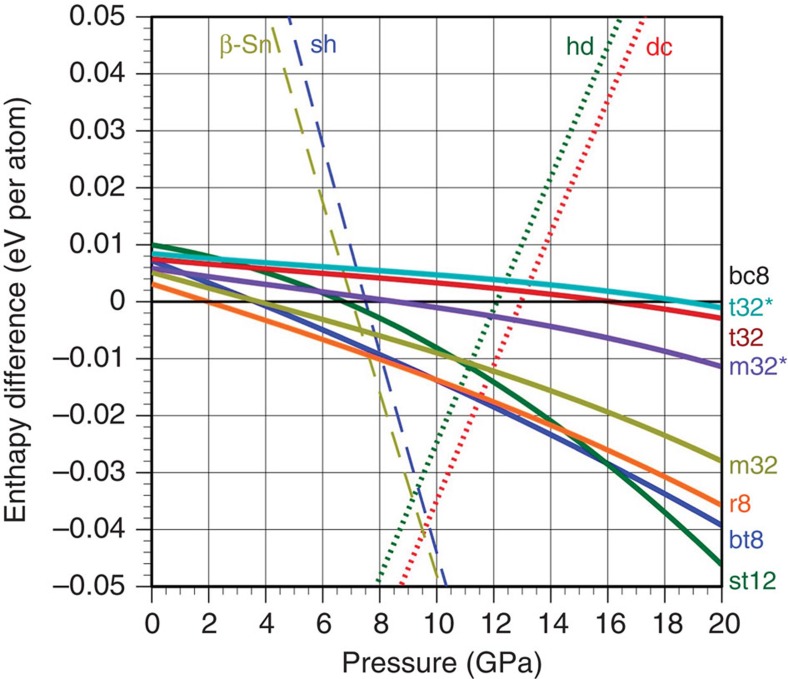
Enthalpy versus pressure curves for various Si allotropes. Development of the enthalpy difference of the metastable Si phases relative to bc8-Si with pressure calculated within DFT. For completeness, the equilibrium dc-Si, (β-Sn)-Si and sh-Si and the metastable hd-Si are shown, whereby the latter follows the dc-Si trend closely^43^.

Why is this? In the ‘near-equilibrium' DAC case, the phase transformations that occur are constrained by equilibrium thermodynamics, as well as kinetic barriers to specific metastable phase formation on depressurization. We therefore suggest that the observed tetragonal phases arise as a direct result of the higher pressure range accessible due to confinement compared with free-surface irradiation, as well as the ultra-rapid depressurization and rapid thermal quenching following the confined microexplosion event. We propose that these tetragonal phases may result from a final transformation from metallic phases that are denser than the β-Sn Si phase but have a wider pressure and temperature stability range. Under ultra-rapid quenching, transformation from denser metallic phases may result in alternative transformation pathways and the formation of metastable tetragonal phases that are not possible under near-equilibrium transformations from the β-Sn Si phase. Furthermore, comparison of the calculated relative enthalpies of the new tetragonal phases with those of the known metastable phases r8 and bc8, as well as that of dc-Si, hd-Si and the metallic β-Sn and simple hexagonal (sh) phases, is shown in Fig. 5. These data provide information on the expected relative stability of the new phases. All of the newly predicted tetragonal phases are only slightly less energetically favourable than bc8 and r8 at 0 GPa but show interesting trends with increasing pressure in which bt8 and st12 follow the trend of r8, but both tetragonal phases with 32 atoms appear to closely follow bc8. The monoclinic structures do not follow either trend closely, which may account for the fact that no strong evidence for their formation was observed experimentally. These calculations are again consistent with the likely metastability of the bt8, st12 and t32 polymorphs at ambient pressure and temperature, particularly if appreciable kinetic energy barriers exist to their further transformation to more stable Si phases at room temperature and pressure.

It should also be noted that depressurization to near-ambient pressure is expected in our particular case even before FIB milling. First, the presence of a void in the capping SiO_2_ layer immediately above the laser-modified zone in Si precludes any substantial confinement (and hence ‘pressure medium') following the laser modification. Note that these voids can be observed prior to ion-milling and their formation is thus not correlated with the thinning process. Second, small residual stresses may be present within the laser-modified material similar to that present in the r8/bc8 phases generated by indentation pressure^38^. Although the newly observed bt8 and st12 polymorphs presumably arise from metallic Si phases more dense than the (β-Sn)-Si, and hence must originate from transient pressures above ∼15 GPa, the presence of the void will again ensure that the level of residual pressure or stress within the laser-modified zone in Si is limited to a few GPa at most.

Such metastable phases of Si are expected to be greatly advantageous since they enable simple ‘pressure-induced bandgap engineering' for innovative future applications. For example, the known narrow bandgap r8 polymorph is predicted to possess a significantly larger light absorption overlap with the solar spectrum than standard dc-Si^6^. Similarly, the bc8 phase in the form of a hydrogenated nanoparticle is expected to enable multiple excitation generation and thus more efficient next-generation photovoltaics^39^. Moreover, a very recent study identified a new allotrope of Si that was not directly formed through the polymorphism of Si itself, but via high-pressure synthesis from a precursor^40^. This novel Si structure was shown to possess a quasi-direct bandgap and is thus expected to be very useful for thin-film solar applications^40^. Indeed, a number of further Si structures with (quasi)direct bandgaps for future photovoltaic devices^41^,^42^ and other useful electronic properties have been predicted^4^,^43^. Such structure prediction has become an immensely powerful tool in the search for novel, useful materials. It can, however, not predict how such structures, once identified, can be synthesized. This clearly is the strength of our current study where the experimental synthesis method of novel metastable phases is akin to the computational modelling itself and may also enable pathways for the synthesis of many useful new phases from other elements and compounds.

Thus, having observed several new tetragonal metastable phases, we can consider their possible properties. According to our calculations, bt8 has a density of 2.73 g cm^−3^ (17% higher than dc-Si), t32 of 2.55 g cm^−3^ (9% higher than dc-Si) and st12 of 2.47 g cm^−3^ (6% more dense than dc-Si). With the exception of the hd-Si polymorph, st12-Si possesses the lowest density of all metastable polymorphs, while t32 exhibits the same density as the well-known bc8 and r8 polymorphs and bt8 is considerably more dense. Consequently, the calculated electronic structure of bt8 suggests metallic behaviour, with a low density of states at the Fermi level. However, accounting for the well-known underestimation of the bandgap by DFT methods^44^, it is likely that it is a narrow bandgap semiconductor. Thus, bt8-Si might also be a good material for solar cells, including its use in nanoparticle form for multiple exciton generation in next-generation photovoltaics applications similar to the semimetallic bc8 phase. In addition, the narrow bandgap would also improve Si absorption in the infrared and hence open up applications for improved mid-infrared detectors. The relatively low-density st12-Si phase has been predicted to be an indirect bandgap semiconductor with a bandgap energy between 1.1 eV (ref. 34) and 1.67 eV (ref. 4). Our calculated bandgap is consistent with these previous reports. Furthermore, the calculated band structure and density of states suggest that st12-Si may be a good candidate for a superconductor (*T*_c_ around 35 K) when sufficiently doped^4^. In addition, comparison with a related metastable system, germanium, would suggest a high thermal stability of st12-Si. While the r8 and bc8 phases of Si are stable to ∼200 °C (refs 5, 45), the same structures formed from Ge are not stable at room temperature^46^. In contrast, however, the st12-Ge structure is stable to at least 200 °C (refs 35, 45). If the metastable Si phases follow a similar trend, st12-Si can be expected to be thermally stable to above 400 °C. This clearly would be highly beneficial for any industrial processing of this interesting phase. In terms of the Si-VIII and t32 phases, in the absence of definitive theoretical predictions of their band structure, it is not yet possible to speculate on possible applications until such time as their properties are calculated or measured. Nevertheless, our technique of laser-induced microexplosion for the production of zones of these new material phases (confined within a bulk substrate) lends itself to such further study of their properties, as well as exploiting any attractive applications that emerge.

In summary, for the first time we have produced two new metastable tetragonal phases of Si within the bulk of the material in a form that allows their further study. These new phases are likely to exhibit interesting properties, such as a predicted low-bandgap semiconductor (bt8-Si) and potentially superconducting behaviour (st12-Si). In addition, we observe several tetragonal structures with 32 atoms in the unit cell, Si-VIII and two other candidate structures that warrant further study to measure properties and potential applications. Furthermore, the microexplosion technique that we use is able to produce ordered volumes of these new materials in patterned arrays within the bulk that can allow exploitation of attractive material properties. Finally, we have shown that ultra-rapid depressurization and thermal quenching from a microexplosion-induced plasma within a solid can lead to novel non-equilibrium phase transformation pathways that can open up a range of new metastable end phases to expand the search for novel material properties.

## Methods

### Laser irradiation conditions

The microexplosion experiments were performed with 170 fs, 790 nm, 0.1–1.0 μJ single-laser pulses tightly focussed by a × 150-high-numerical aperture (NA=1.45) microscope objective through immersion oil as a refractive index-matching liquid (*n*=1.515) onto a Si surface buried under a 10-μm-thick SiO_2_ layer, which provides the required confinement. The focal spot, measured using the knife-edge technique, was 0.74 μm in diameter (full-width at half-maximum)—see Supplementary Fig. 2 and Supplementary Note 1. As the intensity in the focal spot is rather high, we reduced the pulse energy below the ∼1-nJ level and used a sharp edge of an etched Si window covered by Si_3_N_4_ film. The pulsed laser repetition rate was kept at 1 kHz. The sample was a commercially available thermally oxidized Si wafer with a 10-μm-thermally grown SiO_2_ layer. The sample was placed on a computer-controlled nanopositioning stage (Nano-Bio200 from MSL Inc.) with 0.4 nm resolution and 2 nm position repeatability, which was moving with a speed of 2 mm s^−1^ to guarantee single-shot microexplosion conditions with the modified zones and voids forming at a distance of 2 μm apart.

As Si is not transparent at this wavelength, the condition of confinement was realized by focusing the laser pulses through the 10-μm transparent SiO_2_ layer onto the underlying Si wafer. This allowed for the delivery of up to ∼2 × 10^14^ W cm^−2^ onto the Si surface.

### Confined microexplosion

At the intensity of the ultrafast laser pulse above the ionization threshold at ∼10^12^ W cm^−2^, both Si and SiO_2_ convert into a plasma state at the beginning of the laser pulse. The density of free electrons becomes significant due to multiphoton and electron impact ionization processes. It reaches the optical breakdown threshold^47^: 
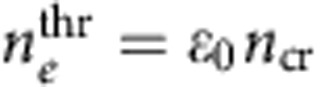
, here *ɛ*_0_=13.7 at 800 nm, and *n*_cr_ is a critical plasma density, 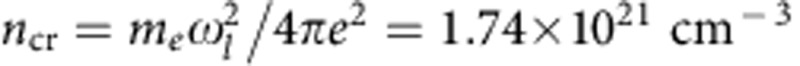, *m*_*e*_ and *e* are, respectively, the electron mass and the charge, and *ω*_*l*_=2.356 × 10^15^ s^−1^ is the laser frequency at 800 nm. At the optical breakdown (ionization) threshold, the real part *ɛ*_re_ of the dielectric function *ɛ*=*ɛ*_re_+*iɛ*_im_ changes from positive, through zero and into the negative range^47^, and the absorption of laser light occurs at the skin layer of the plasma. The real and imaginary parts of the complex refractive index *N=n+ik* are 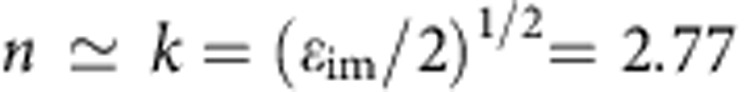 and the absorption coefficient. The thickness of the skin layer, where the laser field decreases *e*-fold and where almost all the absorbed laser energy is deposited, is *l*_s_=*λ*/4*πk*=23 nm. The energy density in the absorbed volume, at an incoming laser fluence of 48 J cm^−2^ in a focal spot of 0.74 μm in diameter (full-width at half-maximum), reaches up to ∼20 MJ cm^−3^ that immediately converts to a maximum electron pressure of 20 TPa (see Supplementary Figs 2 and 3 and Supplementary Note 2 for details).

### TEM imaging and electron diffraction pattern recording

The voids within the SW-modified areas at the Si/SiO_2_ interface were opened up from the bulk using a dual-FIB system and characterized by scanning electron microscopy (SEM)—see Fig. 1. A FEI Helios 600 NanoLab FIB System was used for the milling and the samples were prepared using the ‘H-bar' method^1^—see Fig. 1. SEM imaging was performed using the SEM column of the FIB system. These areas were then thinned to electron transparency (∼100 nm) and investigated by TEM. The TEM was performed using a Philips CM300 operating at an accelerating voltage of 300 keV. Therefore, conventional bright-field imaging, selected area electron diffraction (using an aperture of ∼300 nm diameter) and dark-field imaging were employed. All images and diffraction patterns were taken on photographic negatives, which were then scanned for detailed analysis. The images in Fig. 1c–e were taken within 2 h after the FIB milling. We note that some of the unidentified reflections disappeared from the diffraction patterns taken from the same sites 2 weeks after sample preparation. However, the bt8- and st12-related diffraction spots were retained more than a year after the initial observation.

### Analysis of the diffraction patterns

In an initial step, the d-spacings were determined using the known calibration parameters of the TEM. This was done to identify the dc-Si diffraction rings and positively confirm the spacings as unstressed. For higher accuracy measurements, the known unit cell of dc-Si of *a*=5.431 Å (ref. 48) and these dc-Si rings were then used to calibrate the diffraction pattern to avoid any inaccuracies, through offsets from the eucentric height, for example. This calibration was used to determine the d-spacings of all the reflections not attributed to dc-Si. To positively identify a reflection as belonging to a certain phase, we used SingleCrystal (CrystalMaker Software Ltd). Thereby, not only the d-spacings but also the positions of the diffraction spots within an electron diffraction pattern for a given Si phase were simulated and compared with our experimental observations on the SADPs with the aim to find a match. For the known metastable Si structures, .cif files were collected from a database^2^. Using the software, we simulated the electron diffraction patterns of individual crystals of these Si phases exposed to an electron beam, in the same manner as the recorded bright diffraction spots on the negative film in the TEM. Initially, a single crystal of a given phase was simulated, but we then combined in our analysis two to three different single crystals of one metastable phase diffracting along different planes. The thus simulated pattern was superimposed over the observed diffraction patterns to find the coincidence of the simulated reflections with the observed reflections by changing the orientation of the crystal along each particular crystal lattice. A stereographic projection (a two-dimensional representation of a three-dimensional arrangement of planes, plane normals and vectors in a particular crystal) was used of each Si crystal plane. This was necessary since the different tetragonal phases exhibit many overlapping reflection conditions. Consequently, such a ‘single-crystal' approach was essential to confirm to which particular crystal (and hence phase) a certain reflection belongs.

To verify this approach, we tested it against SADPs obtained from Si regions that had been locally modified through point (indentation) loading and exhibit similar metastable phases as a result^3^,^4^. Although the pathway to their formation is fundamentally different, they also consist of nanocrystals of ∼10–30 nm in size, and the SADPs had been obtained under the exact same experimental conditions in the same TEM. Our analysis clearly revealed the presence of two to three metastable crystals in the body-centered cubic bc8 structure as would have been expected in this case. This successful method was then applied to our SADPs. We were able to reproduce the experimental results through such direct comparison and measurement on the observed diffraction images (see Figs 3 and 4).

### Description of structure prediction

AIRSS was employed^21^,^22^ to identify candidates for the structures. In this approach, crystal structures are generated through the repeated full relaxation of randomly created initial structures, within the DFT. We employed the CASTEP 8.0 plane-wave basis set code^32^ with the Perdew–Burke–Ernzerhof^49^ generalized gradient approximation used as functional. The pseudopotentials used were the CASTEP 8.0 default on-the-fly ultrasoft potentials. A k-point sampling of 0.03 × 2*π* Å^−1^ and a plane-wave cutoff of 400 eV were used. The searches encompassed structures with up to 32 atoms in the unit cell. Only structures with enthalpies similar to the known existing metastable phases of Si were considered further.

## Additional information

**How to cite this article:** Rapp, L. *et al.* Experimental evidence of new tetragonal polymorphs of silicon formed through ultrafast laser-induced confined microexplosion. *Nat. Commun.* 6:7555 doi: 10.1038/ncomms8555 (2015).

## Supplementary Material

Supplementary Information Supplementary Figures 1-3, Supplementary Table 1-6, Supplementary Notes and Supplementary References

Supplementary Movie 1 3D animation of bt8-Si crystal structure

Supplementary Movie 2 3D animation of st12-Si crystal structure

Supplementary Movie 3 3D animation of t32-Si crystal structure

Supplementary Movie 4 3D animation of t32*-Si crystal structure

Supplementary Movie 5 3D animation of m32-Si crystal structure

Supplementary Movie 6 3D animation of m32*-Si crystal structure

Supplementary Data 1 cif file for bt8-Si crystal structure

Supplementary Data 2 cif file for st128-Si crystal structure

Supplementary Data 3 cif file for t32-Si crystal structure

Supplementary Data 4 cif file for t32*-Si crystal structure

Supplementary Data 5 cif file for m32-Si crystal structure

Supplementary Data 6 cif file for m32*-Si crystal structure

## Figures and Tables

**Table 1:** Various polymorphs of Si uncovered in the AIRSS searches.

**Label**	**Space group**	**Lattice parameters (Å)**	**Number of atoms**	**V per atom (Å**^**3**^)	**dH (eV per atom)**
dc-Si, Si-I	*Fd-3m*	*a*=5.468	8	20.441	−0.156
hd-Si, Si-IV	*P63/mmc*	*a*=3.850, *c*=6.364	4	20.426	−0.146
bc8-Si, Si-III	*Ia-3*	*a*=6.658	16	18.443	0
r8-Si, Si-XII	*R-3*	*a*=9.440, *c*=5.661	24	18.205	0.003
bt8-Si	*I4*_*1*_*/a*	*a*=6.676, *c*=6.522	16	18.169	0.007
st12-Si	*P4*_*3*_*2*_*1*_*2*	*a*=5.678, *c*=6.825	12	18.340	0.010
*P-42*_*1*_*c*-32 (t32-Si)	*P-42*_*1*_*c*	*a*=9.408, *c*=6.646	32	18.384	0.007
*P4*_*3*_*2*_*1*_*2*-32 (t32*-Si)	*P4*_*3*_*2*_*1*_*2*	*a =*9.403, *c=*6.655	32	18.388	0.008
*C2*-32 (m32*-Si)	*C2*	*a*=9.390, *c*=6.626, *b*=13.305, *β*=134.81	32	18.351	0.006
*P2*_*1*_*c*-32 (m32-Si)	*P2*_*1*_*c*	*a*=5.763, *c*=9.321, *b*=11.039, *β*=79.98	32	18.249	0.005

AIRSS, *ab initio* random structure searching; DFT, density functional theory; PBE, Perdew–Burke–Ernzerhof.

The lattice parameters, volumes of unit cell per atom and enthalpy differences are taken from the 0-GPa computed structures. The enthalpies were calculated using the PBE model within the DFT and are presented relative to bc8/Si-III. The calculated d-spacings at various pressures in the range 0–16 GPa are presented in Supplementary Tables 1–6. Note that one of the t-32 and m-32 structures, were arbitrarily marked with (*) to distinguish them in the text.

**Table 2:** Crystal lattice.

	**0 GPa**	**2 GPa**	**4 GPa**	**8 GPa**	**16 GPa**
*bt8-Si*					
*a*=*b* (Å)	6.676	6.632	6.592	6.522	6.405
*c* (Å)	6.522	6.447	6.381	6.267	6.079
*st12-Si*					
*a*=*b* (Å)	5.678	5.640	5.605	5.540	5.420
*c* (Å)	6.825	6.734	6.649	6.503	6.277
*P-42*_*1*_*c-32 (t32-Si)*					
*a*=*b* (Å)	9.409	9.335	9.269	9.153	8.959
*c* (Å)	6.646	6.597	6.553	6.473	6.340
*P4*_*3*_*2*_*1*_2-32 (t32*-Si)					
*a*=*b* (Å)	9.403	9.330	9.264	9.148	8.956
*c* (Å)	6.655	6.607	6.562	6.483	6.349

DFT, density functional theory.

Lattice parameters of the tetragonal bt8-Si, st12-Si and two tetragonal structures with 32 atoms at various pressures calculated with DFT.

**Table 3:** Experimental interatomic spacings.

**SADP(a) (Å)**	**SADP(b) (Å)**	**Δ*****d*** **(Å)**	**bt8-Si I4**_**1**_**/a**	**st12-Si P4**_**3**_**2**_**1**_**2**	**t32-Si P-421c-Si**	t32*-Si P4_**3**_**2**_**1**_**2**	**Si-VIII (Å)**	**Unidentified**
**10.80**		**0.3**						**?**
**7.90**		**0.2**						**?**
**5.70**		**0.1**		010				
	**5.07**	**0.07**						**?** (Pair)
**4.80**		**0.06**				200		
**4.62**		**↓**			200			
**4.57**	**4.59**		101					
	**4.38**	**0.05**		101				
**4.00**		**0.04**		110				
**3.88**		**↓**					**3.872**	
	**3.70**							**?**
	**3.39**			111				**?**
**3.30**	**3.32**	**0.03**			220	220		
**3.28**		**↓**	200					
**3.25**								**?**
**3.16**								**?**
**3.09**	**3.09**							**?**
**3.04**	**3.04**							**?**
**2.99**					310	310		
**2.95**								**?**
	**2.90**			012				
	**2.85**			200				
	**2.83**							
**2.73**	**2.73**	**0.02**					**2.728**	
	**2.67**	**↓**	211	201		320	**2.673**	
	**2.54**			210	320			
**2.50**	**2.50**						**2.500**	
	**2.35**		220	211	040	400	*2.281*	
	**2.14**		301	013	420			
	**2.01**		103	220				
**1.98**		**0.01**						**?**
**1.96**	**1.96**	**↓**		113				
**1.92**	**1.92**			221		430		
	**1.82**				510	510	**1.825**	
**1.78**				023				
**1.75**			231	311	520	520		
	**1.72**		132 Or 213	222				
**1.69**	**1.69**			004 Or 123				
**1.64**			400		530	440		
**1.60**				312		530		
**1.58**			004				**1.580**	
	**1.53**		411	321		610		

SADP, selected area electron diffraction pattern.

Interatomic spacings of the tetragonal Si phases observed in our ultrafast laser-induced microexplosion experiments are presented in bold (see Figs 3 and 4). The error values are related to the accuracy of measurements of the positions of the diffraction spots in the SADPs as performed in our case. We present previously determined experimental d-spacings for the tetragonal phase Si-VIII in a separate column^36^. The d-spacings of the diffraction spots observed in our experiments that fit the Si-VIII phase are indicated in bold. The remaining d-spacing, marked in italic, is given for completeness only. The question marks in the last column indicate those measured d-spacings that cannot be attributed to any known phase of silicon.

**Table 4:** Comparison of experimental and refined lattice parameters.

	**CASTEP**	**SADP(a)**	**Δ**	**SADP(b)**	**Δ**
*bt8-Si*					
*a*=*b* (Å)	6.648	6.64	−0.01	6.68	0.02
*c* (Å)	6.461	6.43	−0.03	6.32	−0.03
*st12-Si*					
*a*=*b* (Å)	5.650	5.69	0.04	5.67	0.02
c (Å)	6.764	6.77	0.01	6.79	0.03
*t32-Si*					
*a*=*b* (Å)	9.393	9.39	0.00	9.39	0.00
*c* (Å)	6.642	?	?	?	?
t32*-Si					
*a*=*b* (Å)	9.403	9.45	0.05	9.45	0.05
*c* (Å)	6.655	?	?	?	?

DFT, density functional theory; SADP, selected area electron diffraction pattern.

The lattice parameters of *bt8-Si*, st12-Si, t32-Si and t32*-Si determined from the refinement to the d-spacings measured from SADPs (a,b) are compared with the computed parameters obtained with DFT using CASTEP^32^. To ease comparison, the computed lattice parameters and the deviation therefrom (Δ) at the closest pressure point are given, 1 GPa for bt8-Si, st12-Si and t32-Si and 0 GPa for t32*-Si. Where *c* could not be calculated, it is marked with a ‘?' mark.
