# Supplementary material for: Experimental evidence of new tetragonal polymorphs of silicon formed through ultrafast laser-induced confined microexplosion
Source: Nat Commun. 2015 Jun 29;6:7555. doi: 10.1038/ncomms8555 (PMC4491821; doi:10.1038/ncomms8555)
Supplement: Supplementary Information — Supplementary Figures 1-3, Supplementary Table 1-6, Supplementary Notes and Supplementary References [file ncomms8555-s1.pdf]

Supplementary Figures

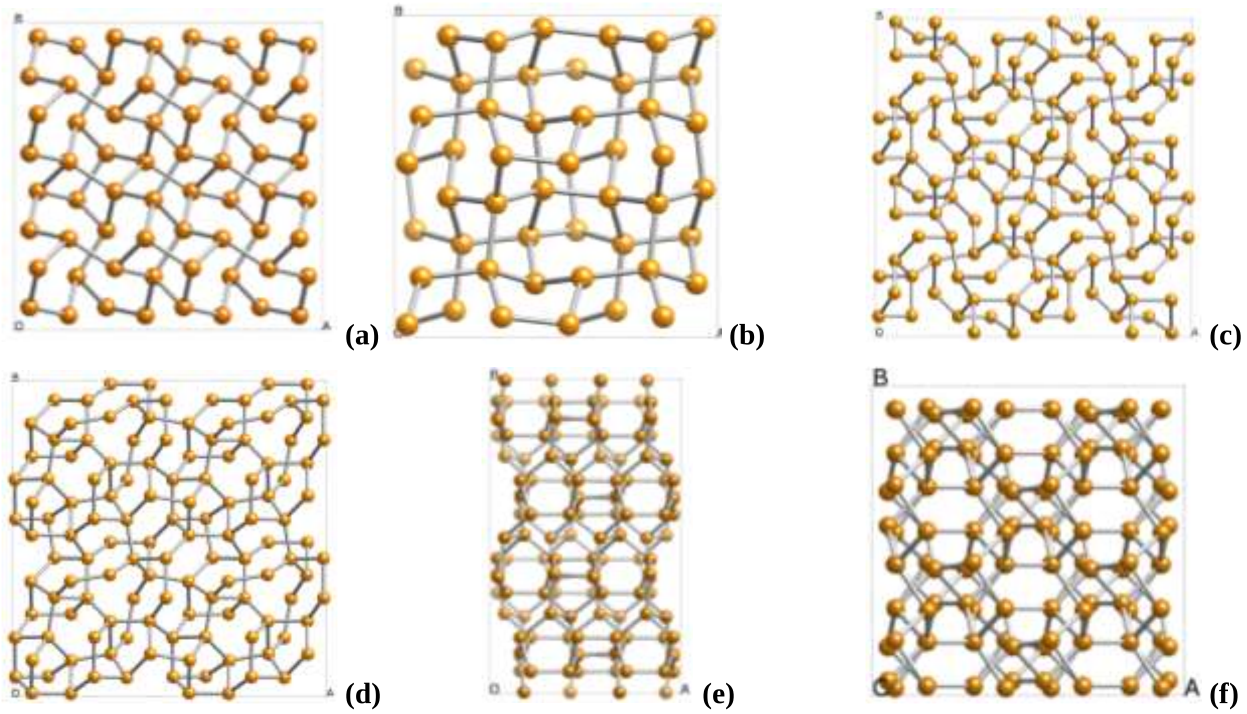

Supplementary Figure 1 | Crystal structure of new phases. The structures are uncovered in the AIRSS searches and the new d-spacings were then calculated using the reflection conditions[1,2] (see Table 4 in the main text). The 3D animations showing different angles of view, prepared with CrystalMaker: (**a**) – bt8-Si (*I41a*), Supplementary Movie 1; (**b**) – st12-Si ($P4_32_12$), Supplementary Movie 2; (**c**) – t32-Si (*P-421c*), Supplementary Movie 3; (**d**) – t32*-Si ($P4_32_12$), Supplementary Movie 4; (**e**) – m32 ($P2_1c$), Supplementary Movie 5; (**f**) – m32*(*C2*), Supplementary Movie 6. The corresponding .cif files for the new phases can be found here:

Suppl-Fig1a_bt8-Si

Suppl-Fig1b_st12-Si

Suppl-Fig1c_t32-Si

Suppl-Fig1d_t32*-Si

Suppl-Fig1e_m32-Si

Suppl-Fig1f_m32*-Si

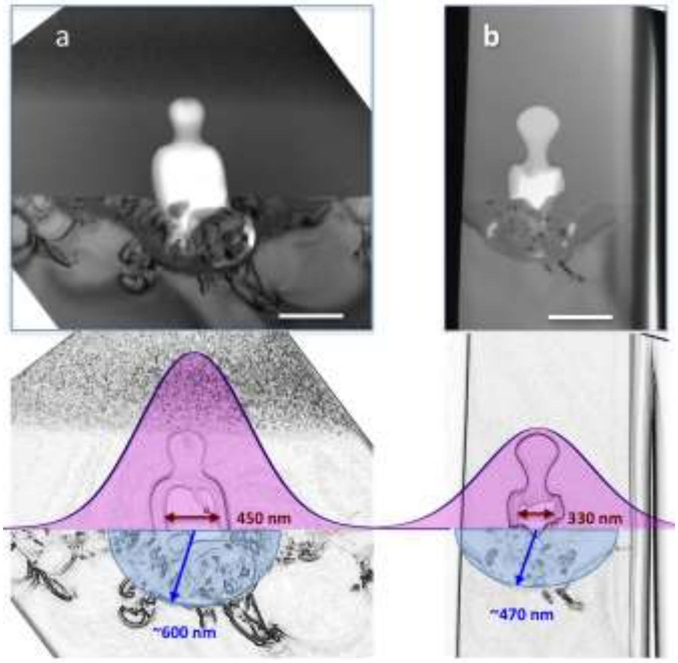

Supplementary Figure 2 | TEM images of the laser-produced voids and shock-wave affected areas. The laser fluences in the experiments were ~95 J cm^{-2} at the laser energy ~440 nJ (**a**) and 60 J cm^{-2} at ~250 nJ (**b**); the scale bars are 500 nm. Below are the same images with tracing contours for better visibility of the areas with indication of the Gaussian shapes of the intensity distribution of the laser intensity on the Si-surface with 740 nm at the FWHM level. The diameter of the voids in SiO_2 are ~450 nm and ~330 nm respectively. The half-ellipsoid contours show the shock-wave affected volume in Si with r_{SW} = ~600 nm (a) and 470 nm (b). The laser pulse is coming from the top.

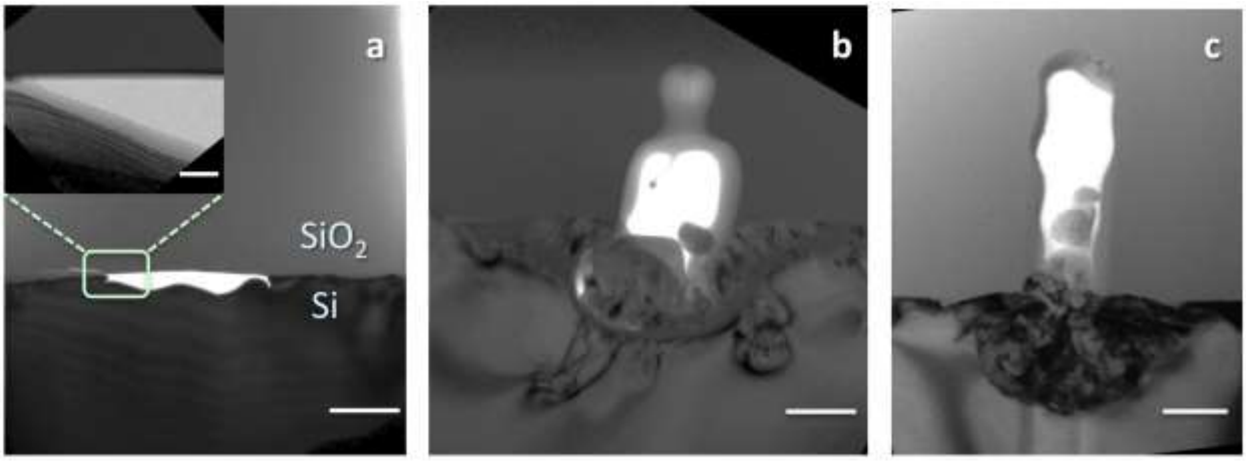

Supplementary Figure 3 | Void formation at various laser fluences. Examples of TEM images of voids formed at the Si/SiO_2 interface at a laser fluence range from 2 J cm^{-2} (**a**) to 95 J cm^{-2} (**b**) to 150 J cm^{-2} (**c**). The shock-wave affected area contains only amorphous Si at 2 J/cm^2, while tetragonal Si-phases were found in the shock-wave affected areas at the laser fluences between 40 J/cm^2 and 150 J/cm^2. The laser pulse is coming from the top, the scale bars are 1 μm and 100 nm in the inset in (**a**), 300 nm in (**b**,**c**).

Supplementary Tables

Supplementary Table 1. D-spacings in [Å] of the tetragonal bt8-Si structure at various pressures calculated using the appropriate reflection conditions[2].

**space group $I4_1/a$ (hkl)**	**0 GPa**	**2 GPa**	**4 GPa**	**8 GPa**	**16 GPa**
101	4.665	4.623	4.585	4.519	4.409
200	3.338	3.316	3.296	3.261	3.203
211	2.715	2.694	2.676	2.644	2.591
112	2.683	2.657	2.633	2.592	2.524
220	2.360	2.345	2.331	2.306	2.265
022	2.333	2.311	2.292	2.259	2.205
301	2.106	2.091	2.078	2.054	2.015
103	2.067	2.044	2.024	1.989	1.932
231	1.781	1.769	1.758	1.738	1.705
132	1.772	1.758	1.745	1.723	1.686
213	1.758	1.740	1.725	1.698	1.654
400	1.669	1.658	1.648	1.630	1.601
004	1.631	1.612	1.595	1.567	1.520
411	1.572	1.561	1.551	1.534	1.505
033	1.555	1.541	1.528	1.506	1.470
114	1.541	1.524	1.509	1.483	1.441
422	1.357	1.347	1.338	1.322	1.296

Supplementary Table 2. D-spacings in [Å] of the tetragonal st12-Si structure at various pressures calculated using the appropriate reflection conditions[2].

**space group *P*4_32_12 (hkl)**	**0 GPa**	**2 GPa**	**4 GPa**	**8 GPa**	**16 GPa**
101	4.365	4.324	4.286	4.217	4.102
110	4.015	3.988	3.964	3.917	3.832
111	3.461	3.432	3.405	3.356	3.271
012	2.925	2.891	2.860	2.804	2.716
200	2.839	2.820	2.803	2.770	2.710
201	2.621	2.601	2.583	2.548	2.488
112	2.600	2.573	2.547	2.502	2.428
210	2.540	2.523	2.507	2.478	2.424
211	2.380	2.362	2.346	2.315	2.261
022	2.183	2.162	2.143	2.109	2.051
013	2.112	2.086	2.061	2.019	1.952
220	2.008	1.994	1.982	1.959	1.916
113	1.979	1.956	1.935	1.897	1.836
221	1.926	1.912	1.899	1.875	1.833
031	1.824	1.811	1.799	1.776	1.736
310	1.796	1.784	1.773	1.752	1.714
023	1.775	1.756	1.739	1.707	1.656
311	1.737	1.724	1.713	1.692	1.653
222	1.730	1.716	1.702	1.678	1.636
004	1.706	1.683	1.662	1.626	1.569
123	1.695	1.677	1.660	1.631	1.584
032	1.655	1.642	1.629	1.606	1.566
312	1.589	1.576	1.564	1.542	1.504
321	1.535	1.524	1.514	1.495	1.462
322	1.430	1.419	1.408	1.389	1.356
133	1.410	1.396	1.384	1.363	1.326

Supplementary Table 3. D-spacings in [Å] of the tetragonal t32-Si structure at various pressures calculated using the appropriate reflection conditions[2].

**space group *P-421c* (hkl)**	**0 GPa**	**2 GPa**	**4 GPa**	**8 GPa**	**16 GPa**
110	6.653	6.601	6.554	6.472	6.335
101	5.428	5.388	5.351	5.285	5.175
210	4.208	4.175	4.145	4.093	4.007
211	3.555	3.528	3.503	3.460	3.387
002	3.323	3.299	3.277	3.237	3.170
102	3.133	3.110	3.089	3.051	2.989
112	2.973	2.951	2.931	2.895	2.835
031	2.836	2.814	2.795	2.760	2.702
131	2.716	2.695	2.676	2.642	2.587
022	2.714	2.694	2.675	2.643	2.588
230	2.609	2.589	2.571	2.539	2.485
122	2.608	2.588	2.570	2.539	2.486
231	2.429	2.410	2.393	2.363	2.313
400	2.352	2.334	2.317	2.288	2.240
222	2.351	2.333	2.317	2.288	2.241
410	2.282	2.264	2.248	2.220	2.173
032	2.281	2.264	2.248	2.220	2.174
330	2.218	2.200	2.185	2.157	2.112
041	2.217	2.200	2.185	2.157	2.112
312	2.217	2.200	2.185	2.158	2.112
411	2.158	2.142	2.126	2.100	2.055
013	2.156	2.141	2.126	2.100	2.057
322	2.052	2.037	2.023	1.997	1.956
241	2.006	1.990	1.976	1.951	1.910
023	2.004	1.989	1.976	1.952	1.911
123	1.960	1.946	1.932	1.909	1.869
430	1.882	1.867	1.854	1.831	1.792
412	1.881	1.867	1.854	1.831	1.792
332	1.845	1.831	1.818	1.795	1.757
341	1.811	1.797	1.784	1.761	1.724
242	1.778	1.764	1.752	1.730	1.693
133	1.777	1.764	1.751	1.730	1.694
233	1.689	1.676	1.665	1.644	1.610
004	1.663	1.650	1.639	1.618	1.585
342	1.637	1.625	1.613	1.593	1.560
104	1.636	1.624	1.613	1.594	1.561
114	1.612	1.600	1.589	1.570	1.538
143	1.590	1.578	1.567	1.547	1.515
024	1.567	1.555	1.545	1.526	1.494
124	1.545	1.534	1.524	1.505	1.474
243	1.526	1.514	1.504	1.485	1.454
224	1.486	1.475	1.465	1.447	1.418
343	1.434	1.423	1.413	1.396	1.367
324	1.402	1.391	1.382	1.365	1.336
044	1.357	1.347	1.338	1.321	1.294
144	1.343	1.333	1.324	1.308	1.281
424	1.304	1.294	1.285	1.269	1.243

800	1.176	1.167	1.159	1.144	1.120
444	1.175	1.167	1.159	1.144	1.120

Supplementary Table 4. D-spacings in [Å] of the tetragonal t32*-Si structure at various pressures calculated using the appropriate reflection conditions[2].

**space group *P4₃2₁2* (hkl)**	**0 GPa**	**2 GPa**	**4 GPa**	**8 GPa**	**16 GPa**
110	6.649	6.597	6.551	6.469	6.333
101	5.432	5.392	5.355	5.289	5.180
111	4.704	4.668	4.636	4.579	4.484
200	4.701	4.665	4.632	4.574	4.478
210	4.205	4.173	4.143	4.091	4.005
021	3.840	3.811	3.784	3.737	3.659
211	3.555	3.528	3.503	3.460	3.388
220	3.324	3.299	3.275	3.234	3.166
012	3.137	3.114	3.093	3.055	2.992
112	2.976	2.954	2.934	2.898	2.838
221	2.974	2.951	2.931	2.894	2.834
310	2.973	2.950	2.930	2.893	2.832
301	2.836	2.814	2.794	2.759	2.702
202	2.716	2.696	2.677	2.645	2.590
311	2.715	2.694	2.675	2.642	2.587
122	2.609	2.590	2.572	2.541	2.488
320	2.608	2.588	2.569	2.537	2.484
321	2.428	2.410	2.393	2.363	2.313
222	2.352	2.334	2.318	2.290	2.242
400	2.351	2.333	2.316	2.287	2.239
032	2.282	2.264	2.249	2.221	2.175
140	2.281	2.263	2.247	2.219	2.172
132	2.217	2.201	2.185	2.158	2.113
041	2.217	2.200	2.184	2.157	2.112
330	2.216	2.199	2.184	2.156	2.111
013	2.159	2.143	2.129	2.103	2.060
411	2.157	2.141	2.126	2.099	2.055
113	2.104	2.089	2.075	2.050	2.007
331	2.103	2.087	2.072	2.046	2.003
322	2.053	2.037	2.023	1.998	1.956
023	2.006	1.991	1.978	1.954	1.914
421	2.005	1.989	1.975	1.951	1.910
123	1.962	1.948	1.934	1.911	1.871
042	1.920	1.905	1.892	1.869	1.830
142	1.881	1.867	1.854	1.831	1.793
340	1.881	1.866	1.853	1.830	1.791
223	1.845	1.832	1.819	1.797	1.760
332	1.845	1.831	1.818	1.795	1.758
033	1.811	1.797	1.785	1.763	1.727
341	1.810	1.796	1.783	1.761	1.724
133	1.778	1.765	1.753	1.731	1.695
422	1.778	1.764	1.752	1.730	1.694
233	1.690	1.677	1.666	1.645	1.611
004	1.664	1.652	1.641	1.621	1.587
440	1.662	1.649	1.638	1.617	1.583
104	1.638	1.626	1.615	1.596	1.563

342	1.637	1.625	1.613	1.593	1.560
114	1.614	1.602	1.591	1.572	1.540
043	1.613	1.601	1.590	1.571	1.538
441	1.613	1.600	1.589	1.569	1.536
143	1.590	1.578	1.567	1.548	1.516
024	1.569	1.557	1.546	1.528	1.496
124	1.547	1.536	1.525	1.507	1.476
423	1.526	1.515	1.504	1.486	1.455
224	1.488	1.477	1.467	1.449	1.419
034	1.470	1.459	1.449	1.431	1.402
314	1.452	1.441	1.431	1.414	1.385
324	1.403	1.392	1.383	1.366	1.338
044	1.358	1.348	1.339	1.322	1.295
144	1.344	1.334	1.325	1.309	1.282
443	1.330	1.320	1.311	1.295	1.268
424	1.305	1.295	1.286	1.270	1.244
434	1.246	1.237	1.228	1.213	1.188
444	1.176	1.167	1.159	1.145	1.121
800	1.175	1.166	1.158	1.144	1.120

Supplementary Table 5. D-spacings in [Å] of the monoclinic m32-Si structure at various pressures calculated using the appropriate reflection conditions[2].

**space group *$P2_1c$*** **(hkl)**	**0 GPa**	**2 GPa**	**4 GPa**	**8 GPa**	**16 GPa**
011	7.076	7.019	6.967	6.876	6.727
100	5.675	5.632	5.591	5.519	5.391
002	5.435	5.396	5.360	5.297	5.194
110	4.848	4.809	4.773	4.710	4.601
012	4.695	4.659	4.627	4.570	4.477
111	4.695	4.658	4.625	4.566	4.466
020	4.661	4.620	4.583	4.566	4.415
102	4.319	4.288	4.260	4.210	4.126
021	4.284	4.247	4.214	4.157	4.126
112	3.919	3.889	3.863	3.816	3.738
120	3.602	3.572	3.545	3.497	3.415
022	3.538	3.509	3.483	3.438	3.364
121	3.538	3.509	3.483	3.436	3.359
013	3.377	3.352	3.329	3.289	3.223
122	3.168	3.143	3.120	3.080	3.014
113	3.134	3.111	3.091	3.054	2.994
031	2.987	2.962	2.938	2.898	2.832
023	2.861	2.861	2.818	2.782	2.724
200	2.838	2.816	2.796	2.759	2.695
211	2.743	2.722	2.703	2.668	2.608
130	2.725	2.702	2.681	2.645	2.583
004	2.718	2.698	2.680	2.648	2.597
202	2.717	2.697	2.678	2.646	2.588
210	2.715	2.693	2.674	2.639	2.578
123	2.708	2.688	2.669	2.636	2.582
032	2.697	2.675	2.654	2.619	2.561
131	2.697	2.675	2.654	2.618	2.558
104	2.636	2.618	2.601	2.571	2.522
014	2.609	2.590	2.572	2.541	2.491
212	2.608	2.589	2.571	2.539	2.484
114	2.537	2.518	2.502	2.473	2.425
132	2.522	2.502	2.483	2.450	2.396
221	2.444	2.425	2.407	2.376	2.322
220	2.424	2.404	2.387	2.355	2.301
213	2.370	2.353	2.337	2.309	2.261
033	2.359	2.340	-	2.292	2.242
024	2.348	2.330	2.313	2.285	2.238
222	2.347	2.329	2.313	2.283	2.233
040	2.330	2.310	2.292	2.260	2.207
124	2.295	2.277	2.262	2.235	2.190
041	2.279	2.259	2.241	2.210	2.159
133	2.271	2.253	2.237	2.208	2.161
223	2.169	2.153	2.138	2.111	2.066
204	2.159	2.144	2.130	2.105	2.063
140	2.156	2.137	2.120	2.091	2.043
042	2.142	2.124	2.107	2.078	2.031

141	2.142	2.123	2.107	2.078	2.030
231	2.108	2.091	2.076	2.048	2.001
214	2.104	2.088	2.075	2.050	2.009
230	2.095	2.078	2.063	2.035	1.988
142	2.051	2.034	2.018	1.991	1.946
034	2.046	2.029	2.015	1.989	1.947
232	2.045	2.029	2.014	1.988	1.944
134	2.010	1.995	1.980	1.956	1.915
043	1.960	1.944	1.929	1.903	1.861
224	1.959	1.945	1.932	1.908	1.869
233	1.924	1.909	1.896	1.871	1.831
143	1.909	1.893	1.879	1.854	1.814
302	1.892	1.878	1.865	1.841	1.800
300	1.892	1.877	1.864	1.840	1.797
311	1.882	1.867	1.854	1.830	1.788
312	1.854	1.840	1.827	1.804	1.764
310	1.854	1.840	1.826	1.803	1.761
241	1.809	1.794	1.781	1.757	1.716
240	1.801	1.786	1.772	1.748	1.708
313	1.778	1.765	1.753	1.731	1.694
321	1.776	1.762	1.750	1.727	1.687
234	1.773	1.76	1.747	1.725	1.689
044	1.769	1.755	1.742	1.719	1.682
242	1.769	1.754	1.741	1.718	1.680
322	1.753	1.739	1.727	1.705	1.664
320	1.753	1.739	1.726	1.704	1.664
144	1.746	1.732	1.719	1.697	1.661
304	1.697	1.685	1.674	1.653	1.619
243	1.689	1.676	1.663	1.653	1.605
323	1.688	1.675	1.664	1.643	1.607
314	1.670	1.658	1.646	1.626	1.592
331	1.634	1.621	1.609	1.588	1.552
332	1.616	1.603	1.592	1.571	1.536
330	1.616	1.603	1.591	1.570	1.534
324	1.595	1.583	1.572	1.553	1.520
244	1.584	1.571	1.560	1.540	1.507
333	1.565	1.553	1.542	1.522	1.489
334	1.490	1.478	1.468	1.450	1.489
341	1.482	1.470	1.459	1.440	1.407
342	1.469	1.457	1.446	1.427	1.395
340	1.469	1.457	1.446	1.427	1.394
402	1.435	1.424	1.414	1.396	1.365
343	1.430	1.419	1.409	1.390	1.359
411	1.423	1.412	1.402	1.384	1.352
400	1.419	1.408	1.398	1.380	1.348
412	1.419	1.408	1.398	1.380	1.349
410	1.403	1.392	1.382	1.364	1.332
413	1.391	1.381	1.371	1.354	1.324
421	1.375	1.365	1.355	1.337	1.307
344	1.372	1.361	1.352	1.334	1.305
422	1.372	1.361	1.352	1.334	1.304
404	1.359	1.348	1.339	1.323	1.294
420	1.357	1.347	1.337	1.320	1.289

423	1.347	1.337	1.327	1.311	1.282
414	1.344	1.334	1.325	1.309	1.281
431	1.306	1.296	1.286	1.270	1.241
424	1.304	1.294	1.286	1.270	1.242
432	1.303	1.293	1.284	1.267	1.238
430	1.291	1.280	1.271	1.254	1.225
433	1.282	1.272	1.263	1.247	1.219
434	1.245	1.235	1.227	1.211	1.185
441	1.225	1.215	1.206	1.190	1.163
442	1.222	1.212	1.204	1.188	1.161
440	1.212	1.202	1.193	1.178	1.150
443	1.204	1.195	1.186	1.171	1.145
444	1.174	1.165	1.156	1.142	1.116

Supplementary Table 6. D-spacings in [Å] of the monoclinic m32*-Si structure at various pressures calculated using the appropriate reflection conditions[2].

space group *C2* (hkl)	0 GPa	2 GPa	4 GPa	8 GPa	16 GPa
001	9.439	9.376	9.320	9.223	9.074
002	4.719	4.688	4.660	4.612	4.537
110	4.698	4.661	4.627	4.567	4.468
111	3.559	3.533	3.509	3.468	3.400
200	3.331	3.306	3.284	3.245	3.181
020	3.313	3.285	3.260	3.214	3.138
003	3.146	-	3.107	3.074	3.025
021	3.126	3.100	3.077	3.035	2.965
112	2.721	2.702	2.685	2.655	2.607
022	2.712	2.690	2.671	2.637	2.581
201	2.615	2.597	2.580	2.551	2.504
004	2.360	2.344	2.330	2.306	2.269
220	2.349	2.330	2.314	2.284	2.234
023	2.281	2.264	2.249	2.222	2.178
113	2.164	2.149	2.136	2.112	2.075
202	2.110	2.095	2.082	2.059	2.023
310	2.105	2.090	2.075	2.050	2.009
130	2.096	2.079	2.063	2.035	1.987
221	2.053	2.037	2.023	1.998	1.957
131	1.957	1.941	1.927	1.901	1.858
024	1.922	1.908	1.896	1.874	1.838
311	1.813	1.800	1.789	1.768	1.734
114	1.783	1.771	1.760	1.741	1.711
222	1.779	1.766	1.755	1.734	1.700
132	1.775	1.761	1.749	1.727	1.690
203	1.752	1.740	1.730	1.711	1.682
400	1.665	1.653	1.642	1.623	1.590
040	1.656	1.642	1.630	1.607	1.569
041	1.632	1.618	1.606	1.583	1.546
133	1.590	1.577	1.567	1.547	1.516
312	1.571	1.560	1.550	1.533	1.504
330	1.566	1.554	1.542	1.522	1.489
042	1.563	1.550	1.538	1.518	1.483
223	1.549	1.538	1.528	1.510	1.482
204	1.492	1.482	1.473	1.457	1.433
420	1.488	1.477	1.467	1.448	1.419
240	1.483	1.471	1.460	1.440	1.407
401	1.472	1.462	1.452	1.435	1.408
043	1.466	1.454	1.443	1.424	1.393
331	1.434	1.423	1.413	1.395	1.366
134	1.419	1.408	1.399	1.382	1.355
241	1.399	1.388	1.378	1.360	1.329
313	1.376	1.366	1.358	1.342	1.318
224	1.361	1.351	1.342	1.327	1.303
044	1.356	1.345	1.336	1.319	1.290
421	1.345	1.335	1.326	1.311	1.285

402	1.308	1.298	1.290	1.276	1.252
332	1.305	1.295	1.286	1.271	1.245
242	1.303	1.293	1.283	1.267	1.240
314	1.218	1.210	1.202	1.189	1.168
422	1.216	1.208	1.200	1.186	1.163
243	1.204	1.195	1.186	1.171	1.147
333	1.186	1.178	1.170	1.156	1.133
440	1.174	1.165	1.157	1.142	1.117
403	1.170	1.162	1.154	1.142	1.121
244	1.109	1.100	1.093	1.080	1.058
423	1.103	1.095	1.088	1.076	1.056
441	1.100	1.092	1.084	1.071	1.048
334	1.081	1.073	1.066	1.053	1.034
404	1.055	1.048	1.041	1.030	1.011
442	1.026	1.019	1.012	0.999	0.979
424	1.005	0.998	0.992	0.980	0.963
443	0.956	0.949	0.942	0.931	0.912
444	0.890	0.883	0.877	0.867	0.850

Supplementary Notes

Supplementary Note 1 | Estimation of maximum pressure, temperature and the cooling and depressurisation time in the laser absorbed volume

We note that the Young's moduli in Si and silica are respectively 165 GPa and 78 GPa. Therefore, shock wave propagation and its termination occur in both materials in different time and space regimes, resulting in significant asymmetry of the void and shock-affected areas. As a result, the expanding shock front is distorted by perturbations due to this asymmetry, also enhanced by hydrodynamic instabilities. Such processes make accurate predictions of the shape and spatial pressure/temperature distribution in the laser/shock affected material very difficult. However, the average size of the void, maximum temperature and pressure, shock-wave propagation distance, cooling and pressure release times can be reasonably estimated on the basis of the energy and mass conservation laws.

Irradiation of the Si-surface in the current experiments with tightly focused 170 fs, 800 nm laser pulses at two energy density levels of F = 60 J cm^{-2} (laser energy 250 nJ) and 95 J cm^{-2} (laser energy 440 nJ) produces the following conditions in the laser-affected Si and SiO_2 presented in Supplementary Figure 2. The ionisation threshold (optical breakdown) is determined by the condition that the real part of dielectric permittivity turns to zero, Re(ε) = 0 as a result of laser excitation.[3,4] The ionisation threshold in Si (0.034 J cm^{-2}) is lower than that in SiO_2 (0.1 J cm^{-2}).[4] Therefore, silicon starts to absorb laser energy earlier in the pulse time. Both Si and O are ionised in a l_{abs} ~23-nm thin layer adjacent to the Si/SiO_2 interface where about A =50% of the laser energy is absorbed.

The absorbed energy density $E_{abs} = 2AF/l_{abs}$ (absorption coefficient and absorption depth, $A \cong 0.5$; $l_{abs} \cong 2.3\times10^{-6}$ cm) constitutes 21 MJ cm^{-3} (F = 60 J cm^{-2}) and 40 MJ cm^{-3} (F = 95 J cm^{-2}), suggesting a maximum electron pressure in the range of 20 – 40 TPa. The major part of the absorbed energy is contained within the SiO_2-volume where the degree of ionisation is ~1-2, i.e. one or two electrons are stripped from the O and Si atoms. Thus, from the conservation of energy, the maximum energy per atom is in the range 150 eV – 300 eV, which corresponds, by taking into account the ionisation losses, to a maximum electron temperature in the range 50-100 eV.

The rate of electron-ion energy transfer, ν_{en}, is inversely proportional to the ion's mass, $\nu_{en} = \nu_{mom}\left(m_e/M_i\right)$, where the momentum exchange rate equals ν_{mom} ~5×10^{15} s^{-1}.[5,6] For this reason O-ions equilibrate their temperature with electrons faster than the Si-ions, $t_O = \left(\nu_{en}^{O}\right)^{-1}$ = 2.1 ps after the pulse, while in Si $t_{Si} = \left(\nu_{en}^{Si}\right)^{-1}$ =3.7 ps (M_O = 16, M_{Si} = 28). Since the O-ions are in motion earlier than the Si-ions, a two-step shock wave emerges from the laser-heated layer at the Si/SiO_2 interface. One may conjecture that the initial shock-wave driven by O in the SiO_2 is the reason for the appearance of the peculiar shape of the void with a bubble-like structure on the top (see Fig.S2).

The shock wave propagates into the bulk, dissipates the energy, and converts to an acoustic wave at a distance where the pressure at the shock front is reduced to the Young's modulus.[7,8] Taking the Young's modulus of SiO_2 as ~75 GPa, this distance is $r_{SW}^{SiO_2}$ ~0.91 µm for 250 nJ and ~1.1 µm for 440 nJ, respectively. The radius of the equivalent sphere is ~0.3 µm (void volume 0.034 µm^3) for 250 nJ and 0.38 µm (void volume 0.063 µm^3) for 440 nJ, which is in close agreement with the volume of the voids in SiO_2 observed in the experiments, ~0.027 µm^3 and ~0.055 µm^3, respectively (see Supplementary Figure 2). Similar estimates for propagation distance in Si yields r_{SW}^{Si} ~680 nm for 250 nJ and ~800 nm for 440 nJ respectively, which is reasonably close to the values observed in experiments.

Let us define de-pressurisation time as a moment when the pressure reduces to the cold pressure, i.e. the Young's modulus in a particular material. Assuming that the shock wave (SW) front velocity quickly reduces to the sound velocity one may estimate that time as r_{stop}/v_{sound}, where r_{stop} is the distance the SW propagates before converting into an acoustic wave. Taking the speed of sound in Si (v_{Si} = 8.5×10^5 cm/s) and in SiO_2 (v_{SiO_2} = 5×10^5 cm/s) one obtains the respective times of ~ 0.08–0.1 ns in Si and 0.17–0.22 ns in SiO_2. The average temperature at that moment can be estimated assuming that a material has a solid-state atomic number density (n_a = 5×10^{22} cm^{-3}) and the recombination took place during shock-wave propagation consuming an energy necessary for making bonds of the order of the ionization potential ~10 eV. The heat capacity is also changing with temperature from 1.5 k_B as for an ideal gas to $3k_B$ of a solid. Thus, it is reasonable to assume that the average temperature at the time the shock wave stops is around 11,000K, and the density is the same order as in the surrounding pristine crystal. The pressure at the moment of termination of the shock wave is equal to the Young modulus of the surrounding pristine material, but the material is still in a partially ionised plasma state with the degree of ionisation ~10%.

The isochoric cooling is a continuous process but the rate of cooling has several stages, each attributed to a particular diffusivity. Each stage is characterised by a different diffusivity coefficient, which is temperature-dependent. The first stage is while the shock wave propagation continues. In this stage cooling from the hot dense plasma state is due to electronic heat conduction with the heat diffusion coefficient, $D = v_e^2/3\nu$, where v_e ~2×10^8 cm s^{-1} is the electron velocity and the collision rate is ν_e ~5×10^{15} s^{-1}, so D = 2.7 cm^2 s^{-1}. Taking the shock-wave propagation distance as L ~800 nm and suggesting that the heat wave moves the same distance, the resulting cooling time is $t_{cool} = L^2/D$ ~1.8 ns and the temperature at the end of the first stage equals ~1.5 eV, qualitatively comparable with the above estimate of the cooling during the shock deceleration and stopping. At 1.5 eV the electronic heat conduction is still efficient. One obtains D ~ 0.24 cm^2 s^{-1} for the second stage when the heat wave propagates further into the bulk. The temperature drops down from 1.5 eV to ~0.19 eV (~2000K) when the heat wave propagates over another 800 nm during 27 ns. The last stage is cooling by conventional linear heat conduction. The drop of temperature from 2000 K to 300 K requires an increase of volume by 6.7 times, which means the heat wave propagates further by ~1.88 µm. Taking the diffusivity in SiO_2 as D_{SiO_2} = 4.76×10^{-3} cm^2 s^{-1}, the time required for propagating this distance and cooling down to room temperature is ~5.2 µs.

The last estimate can be modified for the heat wave propagating in Si. The major difference is in the cooling rate at the third stage because the heat diffusivity in Si is two orders of magnitude higher than that in SiO_2, D_{Si} = 0.5 cm^2 s^{-1}. Hence the cooling time in Si becomes significantly shorter to ~50 ns.

Supplementary Note 2 | Range of laser fluences used for the formation of tetragonal phases of Si

Qualitatively, the level of laser fluence on the Si surface buried under the SiO_2 layer should be sufficient to create a plasma and generate a strong shock-wave with a maximum pressure above the Young's modulus of silicon, Y ~165 GPa. The formation of a void in the Si bulk by the following rarefaction wave is an indication that a maximum pressure above Y has been achieved. In our experimental conditions with single 170 fs laser pulses focused onto a Si surface down to a 0.74 µm (FWHM) diameter spot, the formation of voids commences at a laser pulse energy of ~5 nJ, which generates a fluence of ~2 J cm^{-2}. At this near-threshold laser fluence the shock-wave affected areas are very thin (less then 100 nm thick), and contain only a thin layer of amorphous Si (Supplementary Figure 3a) presumably due to very fast quenching and lacking time for nucleation of any crystal structure.

Further increase of the laser energy demonstrates the extension of voids into the SiO_2 above the Si surface due to the ionisation wave propagation into the SiO_2 – see Supplementary Figure 3. The shock wave also propagates up to 600 nm into the bulk of the Si at a laser fluence of ~150 J cm^{-2} (pulse energy ~600 nJ). Further increase of the laser energy leads to the formation of a void in SiO_2 further and further away from the Si surface. This results in reduced shock-wave propagation into the Si. Analysis of the shock-wave affected areas in Si from TEM images indicate that the new tetragonal phases bt8-Si and st12-Si, as well as t32-Si were found at a specific range of laser fluences from ~40 J cm^{-2} to 150 J cm^{-2} (pulse energy ~100 nJ – 600 nJ).

Supplementary References

1. Pickard C. J., and Needs R. J. Ab initio random structure searching. *J. Phys.: Condens. Matter* **23**, 053201 (2011).
2. Aroyo M. I., Perez-Mato J. M., Capillas C., Kroumova E., Ivantchev S., Madariaga G., Kirov A., Wondratsche H., "Bilbao Crystallographic Server I: Databases and crystallographic computing programs, *Z. Krist.* **221**, 15-27 (2006). doi:10.1524/zkri.2006.221.1.15.
3. Sokolowski-Tinten, K. and von der Linde, D. Generation of dense electron-hole plasmas in silicon, *Phys. Rev. B* **61**, 2643-2650 (2000).
4. E. G. Gamaly and A. V. Rode, Transient optical properties of dielectrics and semiconductors excited by an ultrashort laser pulse, *Journ. Opt. Soc. Am. B* **31**, C36-C43 (2014).
5. P. B. Allen. Theory of thermal relaxation of electrons in metals. *Phys. Rev. Lett*. **59** 1460-1463 (1987).
6. E. G. Gamaly, A. V. Rode, Physics of ultra-short laser interaction with matter: From phonon excitation to ultimate transformations, *Progress in Quantum Electronics* **37**, 215-323 (2013).
7. Juodkazis, S., Misawa H., Gamaly E. G., Luther-Davies B., Hallo L., Nicolai P., Tikhonchuk V. T. Laser-induced microexplosion confined in the bulk of a sapphire crystal: Evidence of multimegabar pressures. *Phys. Rev. Lett*. **96**, 166101 (2006).
8. Gamaly E. G., Juodkazis S., Misawa H., Luther-Davies B., Hallo L., Nicolai P., Tikhonchuk V. T., Laser-matter interaction in the bulk of a transparent solid: Confined microexplosion and void formation. *Phys. Rev. B* **73**, 214101 (2006).
